# Prediction Analysis of Integrative Quality Zones for *Corydalis yanhusuo* W. T. Wang Under Climate Change: A Rare Medicinal Plant Endemic to China

**DOI:** 10.3390/biology14080972

**Published:** 2025-08-01

**Authors:** Huiming Wang, Bin Huang, Lei Xu, Ting Chen

**Affiliations:** 1 Sino-Pakistan International Center on Traditional Chinese Medicine, School of Pharmaceutical Sciences, Hunan University of Medicine, Huaihua 418000, China; shiyun08262025@163.com; 2 The First School of Clinical Medicine, Yunnan University of Chinese Medicine, Kunming 650500, China; huangbin@ynucm.edu.cn (B.H.); xulei@ynucm.edu.cn (L.X.)

**Keywords:** *Corydalis yanhusuo* W. T. Wang, ArcGIS, MaxEnt, potential geographic distribution, integrative quality zones

## Abstract

*Corydalis yanhusuo* W. T. Wang, commonly known as Yanhusuo, is an important and rare medicinal plant resource in China. Its habitat integrity is facing severe challenges due to climate change and human activities. Establishing an integrative quality zoning system for this species is of significant practical importance for resource conservation and adaptive management. This study integrates multiple data sources, including 121 valid distribution points, 37 environmental factors, future climate scenarios (SSP126 and SSP585 pathways for the 2050s and 2090s), and measured content of tetrahydropalmatine (THP) from 22 sampling sites. A predictive framework for habitat suitability and spatial distribution of effective components was constructed using a multi-model coupling approach (MaxEnt, ArcGIS spatial analysis, and co-kriging method). The results indicate that the MaxEnt model exhibits high prediction accuracy (AUC > 0.9), with the dominant environmental factors being the precipitation of the wettest quarter (404.8~654.5 mm) and the annual average temperature (11.8~17.4 °C). Under current climatic conditions, areas of high suitability are concentrated in parts of Central and Eastern China, including the Sichuan Basin, the middle–lower Yangtze plains, and coastal areas of Shandong and Liaoning. In future climate scenarios, the center of suitable areas is predicted to shift northwestward. The content of THP is significantly correlated with the mean diurnal temperature range, temperature seasonality, and the mean temperature of the wettest quarter (*p* < 0.01). A comprehensive assessment identifies the Yangtze River Delta region, Central China, and parts of the Loess Plateau as the optimal integrative quality zones. This research provides a scientific basis and decision-making support for the sustainable utilization of *C. yanhusuo* and other rare medicinal plants in China.

## Simple Summary

*Corydalis yanhusuo* W. T. Wang is a valuable and increasingly scarce Chinese medicinal plant threatened by climate shifts and human activities. To help protect it and guide sustainable cultivation, this study mapped areas where it grows best now and where it might thrive in the future under changing climates. Researchers combined data on where the plant is found, key environmental conditions like rainfall and temperature patterns, and measurements of its key medicinal compound (THP) from different locations. They found that the plant currently grows best in specific regions of Central and Eastern China. However, as the climate warms, these suitable growing areas are predicted to shift northwestward. This study also identified which specific climate factors influence the levels of the plant’s beneficial medicinal compound. Based on all this information, the research pinpointed optimal zones across several Chinese provinces (including Shanghai, Zhejiang, Anhui, Hubei, Henan, Ningxia, Shaanxi, Gansu, Hunan, Jiangxi, and Shanxi) for cultivating high-quality *C. yanhusuo*. These findings provide crucial guidance for farmers on where to grow this important medicinal plant effectively and for conservationists working to protect its wild populations, ensuring its availability for future generations.

## 1. Introduction

*Corydalis yanhusuo* W. T. Wang, a perennial herbaceous plant belonging to the *Papaveraceae* family and the *Corydalis* genus, has its medicinal parts primarily in the tubers. This species is predominantly found in the provinces of Zhejiang, Jiangsu, and Anhui in China [1]. As a traditional analgesic, *C. yanhusuo* has been utilized in clinical practice in China for thousands of years [2]. Numerous studies have demonstrated its diverse pharmacological effects, including cardiovascular protection [3], anti-tumor [4], antioxidant [5], anti-inflammatory [6], and neuroprotective properties [7]. The therapeutic efficacy of *C. yanhusuo* is attributed to its rich array of active constituents, including alkaloids [8], polysaccharides [5], and pentacyclic triterpenoids [9]. Among these, alkaloids are considered the primary bioactive compounds [10], with tetrahydropalmatine (THP) being particularly notable [11]. THP exhibits significant analgesic [12], sedative [13], cardiovascular protective [14], anti-inflammatory [6], and anti-tumor effects [4]. Currently, THP remains a key indicator for assessing the quality of *C. yanhusuo* as per the Chinese Pharmacopoeia [2,11]. Therefore, accurately determining the content of THP is crucial for evaluating the medicinal efficacy and quality control of *C. yanhusuo*.

Despite its strong reproductive potential, *C. yanhusuo* shows a significant dependence on specific habitat conditions. With the degradation of habitats in modern China, the natural habitats of wild *C. yanhusuo* have been continuously shrinking, leading to a reduction in its suitable distribution area. Currently, wild populations are endangered due to environmental problems [15]. The drastic decline in population numbers and the significant contraction of their geographical range not only jeopardize the species’ survival but also pose a severe challenge to the sustainable utilization of traditional Chinese medicinal resources. In 2020, this species was included in the “Red List of Biodiversity in China—Higher Plants,” with an assessment status of Vulnerable (VU) [16]. Consequently, a systematic analysis of the potential geographical distribution patterns of *C. yanhusuo* in China is not only valuable for ecological research but also urgently needed for the conservation and sustainable development of medicinal plant resources.

The geographical distribution of a species reflects its long-term adaptation to environmental factors, illustrating the complex interactions between the species and its habitat. As a typical temperate plant, the growth of *C. yanhusuo* has specific requirements regarding soil, climate, and nutrient management. These factors collectively determine its optimal growth conditions and the efficient synthesis of alkaloids [17]. Traditional field surveys are indispensable for monitoring species distribution and subtle changes in habitat [18]; however, they often struggle to provide comprehensive and continuous spatial coverage data. Consequently, in the context of global climate change, traditional methods have limitations in accurately predicting dynamic species distributions. Species distribution models (SDMs), including the Maximum Entropy model (MaxEnt), utilize species occurrence data and ecological factors, primarily environmental variables, to predict suitable habitats and elucidate biodiversity distribution patterns [19]. As a leading SDM tool, MaxEnt operates efficiently with presence-only data, offering high predictive accuracy even with limited sample sizes, a feature that underpins its widespread adoption in ecological research [20,21]. Currently, it is integrated with chemical constituents and climate variables to assess climate impacts on medicinal plant quality and identify optimal cultivation areas [22,23].

This study focuses on the medicinal plant *C. yanhusuo*, employing the MaxEnt model to predict the distribution characteristics of its suitable habitats and the impact of climate change. By integrating the co-kriging method and ArcGIS spatial analysis techniques, we investigate the effects of key environmental factors on the accumulation of THP and delineate high-quality cultivation areas. The main research objectives are to (1) predict the current and future climate-suitable habitats for *C. yanhusuo*, (2) identify key environmental factors influencing THP accumulation, and (3) delineate optimal cultivation zones based on habitat suitability and phytochemical quality. Achieving these objectives will provide a scientific basis for the conservation of *C. yanhusuo* resources, artificial cultivation planning, and ecological adaptability research. Furthermore, this work generates concrete recommendations for habitat conservation policies and precision cultivation practices, offering practical guidance for sustainable management of endangered medicinal plants.

## 2. Materials and Methods

### 2.1. Species Data Acquisition and Processing

**Figure 1:**
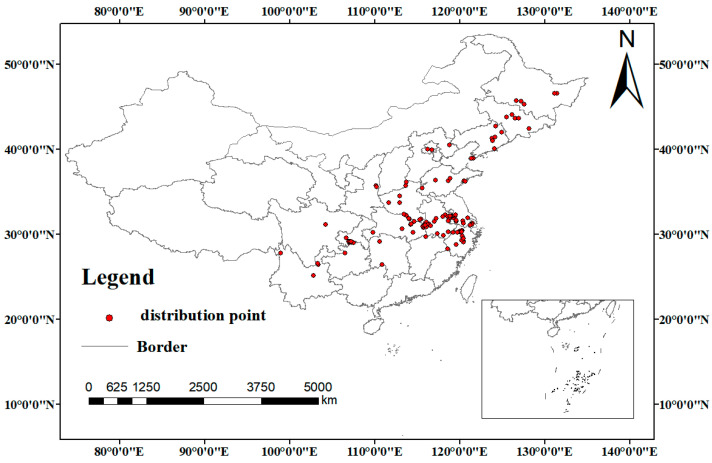
The distribution information of *C. yanhusuo*.

Distribution data for *Corydalis yanhusuo* were aggregated from three biodiversity repositories: the Chinese Virtual Herbarium (CVH; http://www.cvh.ac.cn, accessed on 14 March 2025), the Plant Photo Bank of China (PPBC; http://ppbc.iplant.cn/, accessed on 16 March 2025), and the Global Biodiversity Information Facility (GBIF; https://www.gbif.org/, accessed 19 March 2025). Specimen coordinates were compiled into a CSV file and processed using ArcGIS 10.8 (ESRI, Redlands, CA, USA). To address spatial autocorrelation, the “Spatially Rarefy Occurrence Data for SDMs” tool within SDM Toolbox filtered specimens within 5 km proximity [24]. This refinement yielded 121 spatially independent points for modeling (Figure 1). The tuber’s predominant bioactive compounds are alkaloids, notably tetrahydropalmatine (THP), recognized for its analgesic properties and abundance. As THP quality standards are codified in the Chinese Pharmacopoeia (2025 edition), it served as the primary quality indicator in this study. Literature searches spanning 2014~2024 were conducted via China National Knowledge Infrastructure (CNKI; https://www.cnki.net, accessed on 31 March 2025) and Google Scholar (https://scholar.google.com, accessed on 31 March 2025), targeting publications on *C. yanhusuo* sampling and THP quantification. Following deduplication and data validation, 22 validated THP measurement records were consolidated into the final dataset (Supplementary File).

### 2.2. Environmental Variables Acquisition and Processing

This study incorporated three categories of habitat variables for *C. yanhusuo*: bioclimatic, edaphic, and topographic factors. Bioclimatic parameters (19 variables representing 1970–2000 baseline and future projections) were sourced from WorldClim (http://www.worldclim.org; accessed 28 April 2022) at 2.5-arc-minute resolution. Future climate scenarios employed the BCC-CSM2-MR model—validated for Chinese climate simulations—projecting conditions for the 2050s (2041–2060) and 2090s (2081–2100) under two Shared Socioeconomic Pathways [25]: SSP126 (low carbon emissions) and SSP585 (high emissions, characterized by peak population, limited innovation, and sustained energy demand) [26]. SSP frameworks effectively address projection uncertainties and represent standardized global socioeconomic–climate narratives [27]. Soil and topographic variables originated from the Harmonized World Soil Database (https://www.fao.org/soils-portal; accessed 15 October 2022) and WorldClim (re-accessed 18 October 2022). All global datasets were clipped to China’s boundaries using ArcGIS 10.8. Variable selection proceeded through (1) initial screening via MaxEnt 3.4.4 iterative calculations using occurrence data and contemporary variables, retaining parameters with >0% contribution; (2) spatial extraction of variable values using ArcGIS’ “Sampling” tool; and (3) Pearson correlation analysis in SPSS 26 (IBM, Armonk, NY, USA), removing collinear variables where |r| > 0.8 and contribution rates were negligible [28]. The refined variable set advanced to subsequent modeling stages.

### 2.3. Predictive Modeling Implementation

MaxEnt (v3.4.4) was employed to model 121 *C. yanhusuo* occurrences against 16 environmental predictors. Model parameters included 75% training/25% testing data partition, 10,000 background points, 1,000,000 maximum iterations, and 10-fold cross-validation. Performance was evaluated via the area under the receiver operating characteristic curve (AUC), where values > 0.9 denote high predictive accuracy [29]. Environmental variable contributions were assessed through Jackknife testing and response curve optimization [30]. Output probability surfaces (*p*: 0~1 range) were thresholded at *p* ≥ 0.5 to identify optimal environmental ranges, with peak probabilities defining factor-specific adaptation thresholds [25]. Subsequent processing in ArcGIS 10.8 involved (1) converting ASCII outputs to raster format; (2) applying Spatial Analyst’s Reclassify tool to consolidate predictions; and (3) classifying habitat suitability via Jenks’ natural breaks as being non-suitable at 0~0.1, having low suitability at 0.1~0.3, having moderate suitability at 0.3~0.5, and having high suitability as 0.5~1 [31]. Habitat area estimates were derived by calculating class-specific grid proportions from reclassified attribute tables.

### 2.4. Centroid Migration

Habitat centroids for *C. yanhusuo* across climate scenarios and temporal periods were calculated using ArcGIS 10.8’s spatial statistics “Mean Center” tool. This quantified centroid positions and displacement trajectories, enabling systematic tracking of optimal habitat migration under climate change regimes [32].

### 2.5. Integrative Quality Zonation

Spearman correlation analysis between THP content (22 *C. yanhusuo* samples) and 37 environmental covariates identified key predictors significantly influencing alkaloid accumulation. These screened variables informed a co-kriging interpolation model mapping component spatial distribution [33]. Final quality zonation integrated Suitable Growth Zones with bioactive compound distribution through raster algebra and fuzzy overlay, yielding a synthesized quality region map for *C. yanhusuo*.

## 3. Results

### 3.1. Model Construction and Screening of Dominant Environmental Variables

**Figure 2:**
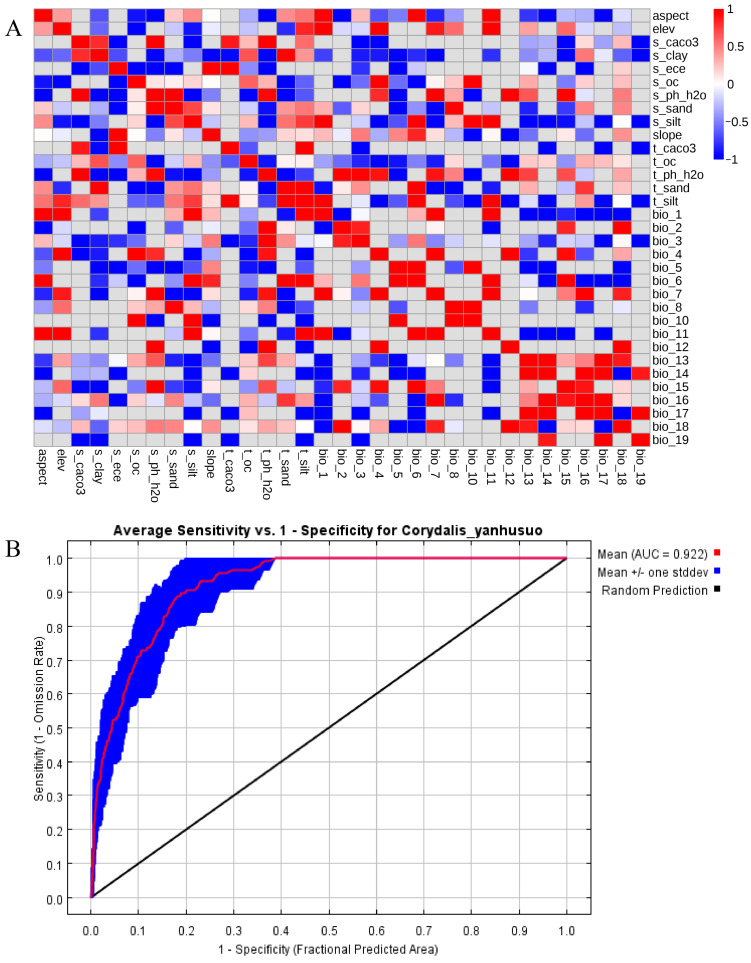
Modeling environmental variables and evaluation of model accuracy. (**A**) Correlation analysis heatmap. (**B**) ROC curve.

Pearson correlation analysis of 33 environmental variables (Figure 2A) informed retention of 16 key variables for MaxEnt modeling (Table 1). Model performance evaluated via the area under the curve (AUC) metric yielded a ROC-derived value of 0.922 (Figure 2B). This exceeds the 0.9 threshold for high predictive reliability, indicating robust discriminative capacity.

**Figure 3:**
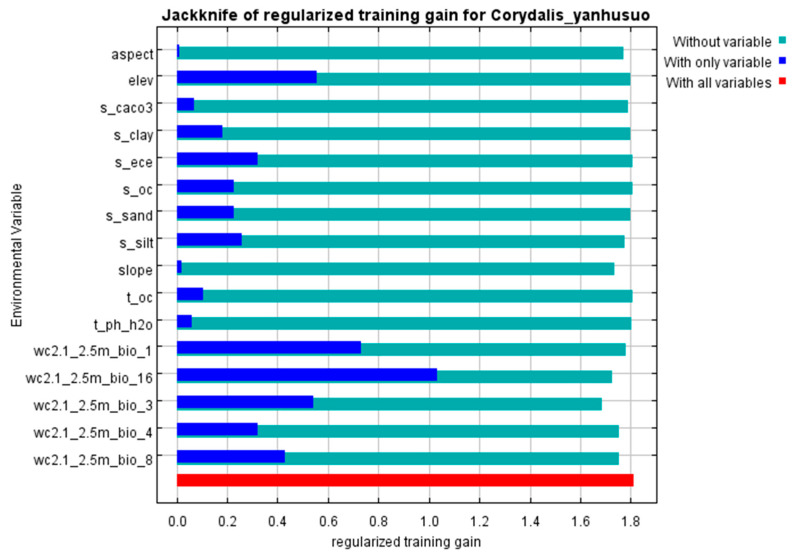
Gain of each variable.

The impact of variables on the distribution model was assessed through contribution percentages and regularized training gains. Under current climatic conditions, four environmental variables (Bio_16, Bio_3, Bio_1, and Bio_4) collectively explain 79.3% of the variance in the model. Among these, Bio_16 contributes the highest proportion (>50%), with all variables exceeding 5% contribution (Table 2). Jackknife tests indicated significant gains (>0.6) for Bio_16 and Bio_1 (Figure 3). Based on their combined contribution percentage and gain values, these two variables were identified as the most critical factors influencing the distribution of *C. yanhusuo*. This result underscores the pivotal role of moisture and temperature conditions in species distribution modeling, suggesting that prioritizing these variables in ecological niche modeling can enhance prediction accuracy, particularly in climate-sensitive regions.

**Figure 4:**
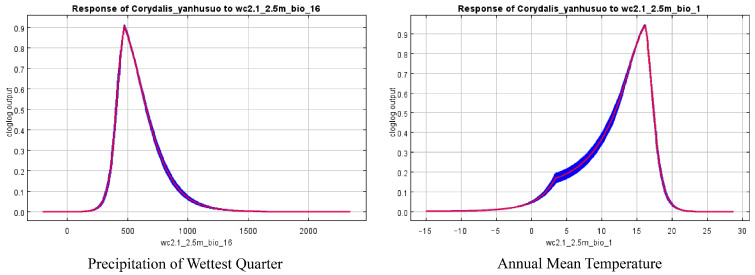
The response curve of dominant environmental variables.

MaxEnt response curves quantified species–environment relationships for *C. yanhusuo*, identifying optimal precipitation and temperature ranges for occurrence probability > 0.5 (Figure 4, Table 3). Precipitation of the wettest quarter and annual mean temperature were the most influential predictors, further confirming their primary role in species distribution.

### 3.2. Spatiotemporal Distribution and Changes in Suitable Habitats of C. yanhusuo

**Figure 5:**
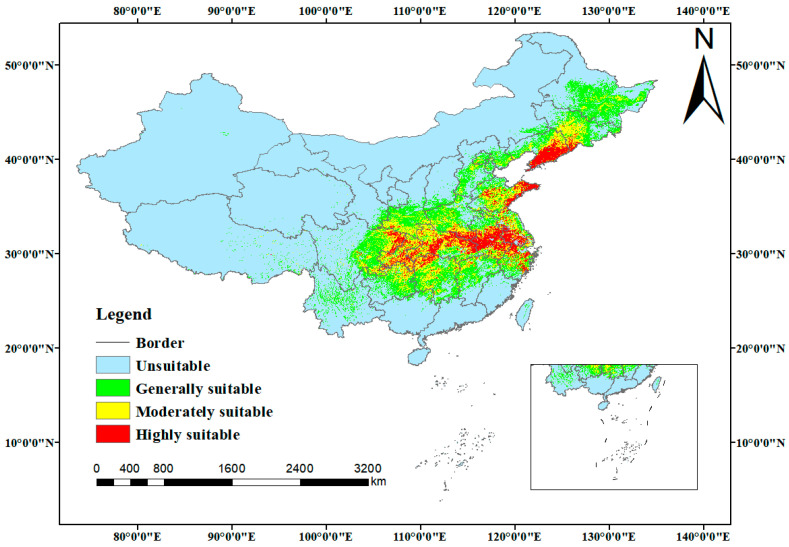
Potential distribution of *C. yanhusuo* under present climate.

Under current climatic conditions, the total area of potential habitat for *C. yanhusuo* is 210.36 × 10^4^ km^2^. Among this, the highly suitable habitats (35.33 × 10^4^ km^2^) are primarily concentrated in China’s Yangtze River basin and eastern coastal regions, including the Sichuan Basin, the middle–lower reaches of the Yangtze River Plain, and coastal areas of Shandong and Liaoning Provinces (Figure 5). The concentrated distribution in these areas indicates that the middle–lower Yangtze and eastern coastal zones represent optimal environments for this species’ growth. The moderately suitable and generally suitable habitats cover 59.52 × 10^4^ km^2^ and 115.51 × 10^4^ km^2^, respectively, with this overall distribution pattern emphasizing the influence of climatic factors on the species’ habitat suitability.

**Figure 6:**
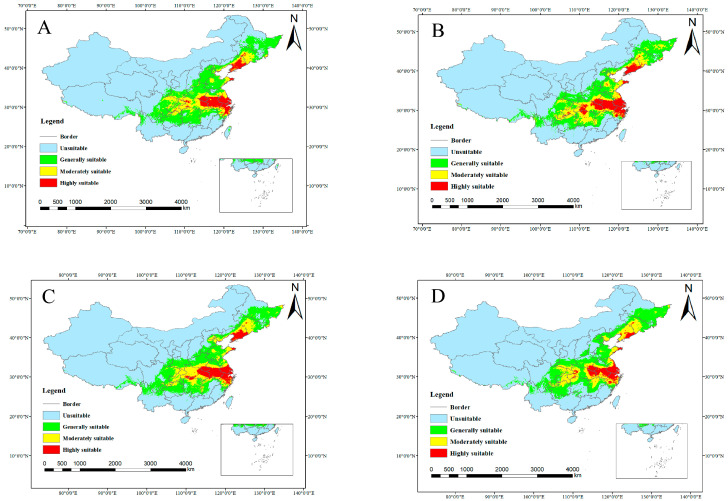
Potential distribution of *C. yanhusuo* under future climate: (**A**) 2041~2060, SSP 126; (**B**) 2081~2100, SSP 126; (**C**) 2041~2060, SSP 585; and (**D**) 2081~2100, SSP 585.

Under the SSP 126 scenario, the distribution of growth-suitable areas shifts markedly over time. For 2041~2060, highly suitable areas (31.18 × 10^4^ km^2^; 3.24% of China) were concentrated in Eastern Coastal and Central China, while moderately suitable areas (55.58 × 10^4^ km^2^; 5.78%) appeared across broader northeastern and central regions. This resulted in a total suitable area of 24.68%. By 2081~2100, highly suitable regions expanded to 37.85 × 10^4^ km^2^ (3.94%), with increased coverage in Northeastern China, and moderately suitable areas grew to 63.76 × 10^4^ km^2^ (6.63%) across diverse regions, yielding a total suitable area of 24.93%. Compared to current conditions, the mid-century period showed reduced high and moderate suitability intensity but a net increase in total coverage. By the late-century period, all suitability categories increased significantly, indicating a progressive expansion and redistribution due to climate influences under this scenario. In the SSP 585 scenario from 2041 to 2060, highly suitable areas (33.77 × 10^4^ km^2^, 3.51% of China’s land area) were concentrated in Eastern and Central China. Moderately suitable areas (60.52 × 10^4^ km^2^, 6.29%) covered broader zones like Hubei, Chongqing, and northern provinces. The total suitable area reached 25.24%. By 2081~2100, highly suitable areas decreased to 26.40 × 10^4^ km^2^ (2.75%), primarily in southeastern, coastal, and central zones, while moderately suitable areas remained substantial (60.29 × 10^4^ km^2^, 6.27%), expanding into central and western areas. The total suitable area declined to 23.82%. Compared to current conditions, the 2050s showed an overall expansion due to gains in moderately suitable areas offsetting losses in highly suitable regions. However, by the 2090s, greenhouse gas emissions drove reductions in all suitability categories, indicating a consistent decline in habitat suitability (Figure 6).

**Figure 7:**
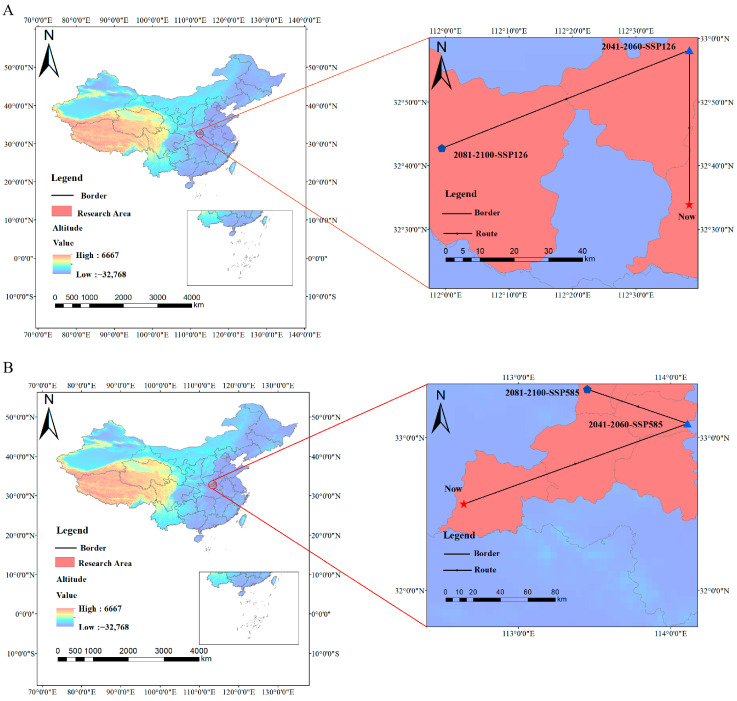
Centroid migration trajectories of *C. yanhusuo*: (**A**) 2050s~2090s, SSP126; and (**B**) 2050s~2090s, SSP585.

Centroid migration analysis reveals distinct shifts in *C. yanhusuo*’s suitable habitat under different climate scenarios. Specifically, the centroid shifts northward by 53.33 km, followed by a southwest movement of 79.88 km under SSP 126, while under SSP 585, it migrates northeast by 177.59 km before moving northwest by 78.96 km (Figure 7; Table 4). Collectively, these trends indicate a northwestward shift of the species’ distribution to higher latitudes with future climate change.

### 3.3. Evaluation of Key Environmental Variables Influencing THP Accumulation and Construction of Integrative Quality Zonation

**Figure 8:**
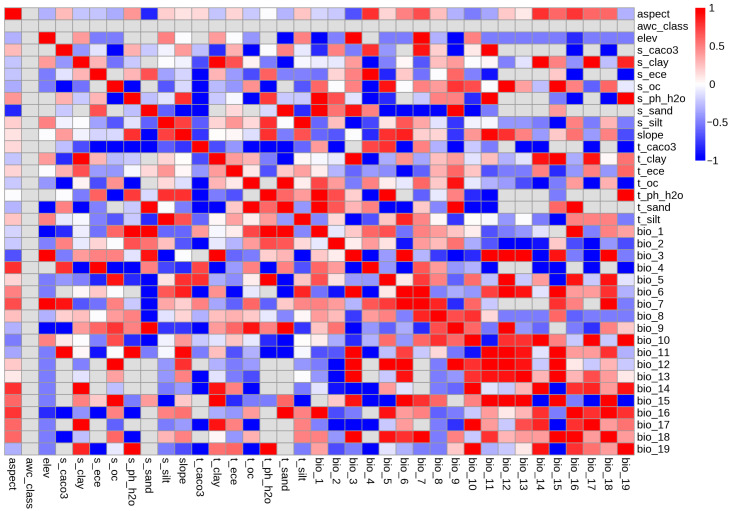
Correlation analysis between THP and environmental variables.

The tetrahydropalmatine (THP) content in *C. yanhusuo* showed significant positive correlations with key temperature variables: mean diurnal range (Bio_2), temperature seasonality (Bio_4), and mean temperature of the wettest quarter (Bio_7) (*p* < 0.01). Higher values in these variables were consistently associated with increased THP accumulation (Figure 8 and Table 5).

**Figure 9:**
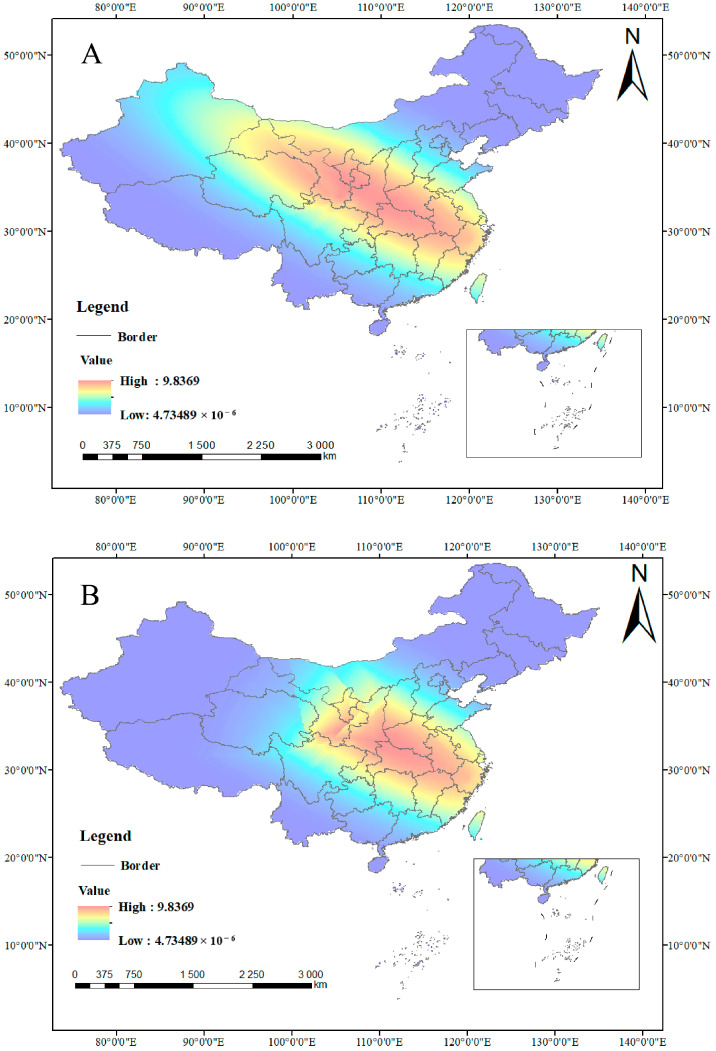
Integrative Quality Zonation. (**A**) Co-kriging analysis of THP. (**B**) Integrated quality regions of *C. yanhusuo*.

The key findings reveal that Bio_2, Bio_4, and Bio_7 are dominant climatic drivers of THP content in *C. yanhusuo*. High THP content is concentrated in Central and Eastern China, with major hotspots in Hubei, Henan, Anhui, and Zhejiang Provinces, indicating optimal environmental conditions for cultivation (Figure 9A). Integration with ecological suitability shows that excellent integrative quality zones are predominantly located in the Yangtze River Delta region, Central China, and parts of the Loess Plateau (Figure 9B), supporting targeted quality-based farming strategies.

## 4. Discussion

This study, based on MaxEnt modeling, reveals that the dominant environmental variables influencing the distribution of *C. yanhusuo* are the Bio_16 and Bio_1. Among these, precipitation has the most significant impact, followed by temperature. Li et al. found, in their research on the plant *Corydalis hendersonii*, that annual mean precipitation is positively correlated with soil nutrients, and that biomass decreases with increasing continentality of rainfall [34]. Additionally, Li et al. employed the MaxEnt method to model *C. hendersonii*, identifying the minimum temperature of the coldest month and annual precipitation as important variables (with a contribution greater than 5%), where precipitation accounted for over 90% of the contribution, indicating its utility in distinguishing habitat suitability [35]. Ali et al.’s MaxEnt modeling also demonstrated that precipitation and temperature variables significantly dominate the distribution of *Corydalis govaniana*, highlighting Bio_16 as a key climatic factor [36]. Collectively, these findings indicate that temperature and precipitation variables have a significant impact on the distribution of *Corydalis* species, which is consistent with our results and reflects the differential responses of species to climate change.

According to the “Flora of China,” *C. yanhusuo* is found in Anhui, Jiangsu, Zhejiang, Hubei, and Henan Provinces, primarily in hilly grasslands. It has also been introduced and cultivated in Shaanxi, Gansu, Sichuan, Yunnan, and Beijing [37]. Model predictions indicate that the current high suitability areas for this species under existing climatic conditions are mainly concentrated in the Yangtze River basin, the Shandong Peninsula, and the Liaodong Peninsula. Specifically, these areas include Chongqing, Sichuan, Liaoning, Shandong, Jiangsu, Anhui, Hubei, Hunan, and Zhejiang, aligning closely with the actual distribution points collected and the range documented in the “Flora of China,” thereby validating the effectiveness of the model. Overall, from the current to the future, the trend of fragmentation in highly suitable habitats is expected to intensify, potentially associated with increased CO_2_ emissions and anthropogenic activities such as overgrazing and land reclamation [38]. Future scenario analyses indicate that under the SSP126 (low emissions) scenario, highly suitable habitats will experience significant contraction by the 2050s, primarily located in the transitional zone between the subtropical regions of Southern and Northern China, with only minimal expansion anticipated by the 2090s. This contraction may result from increased greenhouse gas concentrations, leading to warming in the eastern north subtropical and south subtropical regions, altered water-heat conditions, and the increased frequency of extreme climatic events [39]. In contrast, under the SSP585 (high emissions) scenario, suitable habitats are projected to significantly expand from the present to the 2050s, primarily extending northwest into the plateau climate zone and the temperate regions, with localized contractions expected by the 2090s. The expansion under high-emission scenarios is driven by climate warming, which improves habitat conditions in previously unsuitable plateau and temperate regions. Centroid migration results indicate that the centroids migrate within the boundary between temperate and subtropical regions across different time periods. Specifically, under the SSP126 scenario, the centroid migrates in a north–northwest direction, while under the SSP585 scenario, it exhibits a northeast–northwest trajectory, overall trending northwest. This migration trend may be attributed to two factors: (1) under the backdrop of climate warming, plant distributions generally expand toward higher latitudes and altitudes [40]; and (2) plants of the genus *Corydalis* in China are primarily distributed in the Hengduan Mountains and the Eastern Himalayas [41]. Their embryos require cultivation at low temperatures for more than eight weeks before development can commence [42]. This adaptation to cold environments enhances their survival capabilities in cold habitats.

Plants synthesize a variety of secondary metabolites in response to abiotic stresses such as drought, salinity, ultraviolet radiation, and temperature fluctuations, enabling them to adapt to environmental changes [43,44]. This study found that elevated temperatures significantly enhance the accumulation of tetrahydropalmatine, a secondary metabolite in *C. yanhusuo*. Although direct evidence linking tetrahydropalmatine levels to temperature is lacking, previous research has demonstrated that high temperatures can increase alkaloid content. For instance, after a two-hour high-temperature treatment, the concentration of hydroxycamptothecin in *Camptotheca acuminata* seedlings surged sixfold [45]. Similarly, an average temperature increase of 3 °C during the grain-filling period significantly elevated alkaloid levels in *Lupinus angustifolius* seeds [46]. Conversely, low-temperature stress has been shown to inhibit the biosynthesis of terpenoid indole alkaloids in *Catharanthus roseus* [47]. Collectively, these findings suggest a positive correlation between elevated temperatures and the accumulation of plant alkaloids, which is consistent with the results of this study.

*C. yanhusuo*, recognized as a traditional medicinal herb in Zhejiang Province and one of the “Eight Treasures of Zhejiang,” possesses significant medicinal and economic value. It serves as a crucial pillar for the local herbal industry and a primary source of income for farmers [1]. The main active component, THP, exhibits potent analgesic and sedative effects, demonstrating efficacy in alleviating various types of pain, including chest pain, gastric pain, and dysmenorrhea, earning it the reputation of an “effective analgesic” [48,49]. Modern research has confirmed that THP’s analgesic effects are significant and non-addictive, making it a potential alternative to opioid medications for the treatment of various pain disorders [13,50]. Additionally, its properties of promoting blood circulation and alleviating stagnation reflect the holistic therapeutic characteristics of traditional Chinese medicine, particularly in the treatment of cardiovascular diseases such as coronary heart disease and arrhythmias [50]. However, due to environmental degradation, intensified human activities, and the prolonged growth cycle of *C. yanhusuo*, excessive harvesting has led to a dramatic decline in wild resources, with recovery rates falling far short of demand. To achieve sustainable utilization, comprehensive conservation strategies are urgently needed: First, strict control over the intensity of wild resource harvesting should be implemented to prevent overexploitation. Second, the development of artificial cultivation methods is necessary to meet market demand, with site selection optimized based on the growth habits of *C. yanhusuo*, such as precipitation and temperature requirements. Third, enhancing research support is crucial, including the advancement of fundamental studies, the establishment of germplasm resource banks to preserve genetic diversity, the development of tissue culture techniques to accelerate propagation, the breeding of resilient new varieties (such as those tolerant to high temperatures and drought), and the optimization of harvesting and processing techniques to improve the quality of the medicinal materials.

## 5. Conclusions

This study employs correlation analysis, ArcGIS spatial analysis, and MaxEnt to predict the distribution patterns of suitable habitats for *Corydalis yanhusuo* under current and future climate conditions, while identifying the key environmental factors influencing its distribution. The results indicate that the two primary environmental variables are precipitation of wettest quarter (404.8~654.5 mm) and annual mean temperature (11.8~17.4 °C). Under current climatic conditions, suitable habitats for *C. yanhusuo* are primarily located in regions such as Chongqing, Eastern Sichuan, Southern Liaoning, Eastern Shandong, Southwestern Jiangsu, Central Anhui, Eastern and Central Hubei, Northwestern Hunan, and Northern Zhejiang, covering a total area of approximately 210,360 square kilometers. In future climate scenarios, although the area of suitable habitats is expected to increase slightly, the centroid of distribution will shift northwestward toward higher latitudes. Using co-kriging interpolation to analyze the spatial distribution characteristics of Tetrahydropalmatine, and integrating the predictions of suitable habitats for *C. yanhusuo*, we identify the primary high-quality production areas as being located in Shanghai, Zhejiang, Anhui, Hubei, Henan, Ningxia, Shaanxi, Southeastern Gansu, Northern Hunan, Northern Jiangxi, and Southern Shanxi. These regions are recommended as priority sites for artificial cultivation. Given the sharp decline in wild *C. yanhusuo* resources due to habitat fragmentation and overharvesting, systematic conservation measures are urgently needed. This study proposes a three-tiered protection system: (1) strict control of wild resource harvesting to prevent overexploitation; (2) development of standardized cultivation practices to meet demand, optimizing planting areas based on growth habits (such as responses to precipitation and temperature); and (3) strengthening research support through fundamental studies, establishing a germplasm resource bank to preserve genetic diversity, developing tissue culture rapid propagation techniques, breeding new stress-resistant (heat-tolerant and drought-resistant) varieties, and optimizing harvesting and processing methods to enhance the quality of medicinal materials.

## Figures and Tables

**Table 1:** Environmental variables for MaxEnt model.

Variables	Name	Unit
Aspect	Aspect	/
Elev	Elevation	m
Slope	Slope	◦
Bio_3	Isothermality	/
Bio_1	Annual mean temperature	°C
Bio_4	Temperature seasonality	/
Bio_16	Precipitation of the wettest quarter	mm
Bio_8	Mean temperature of the wettest quarter	°C
T_ph_h2o	Topsoil pH	−log(H^+^)
T_oc	Topsoil organic carbon content	% weight
S_silt	Subsoil silt content	% weight
S_clay	Subsoil clay content	% weight
S_sand	Subsoil sand content	% weight
S_caco3	Subsoil carbonate or lime content	% weight
S_oc	Subsoil organic carbon content	% weight
S_ece	Subsoil electrical conductivity	ds/m

**Table 2:** Environmental variables contributions.

Environmental Variables	Name	Percent Contribution (%)
Bio_16	Precipitation of wettest quarter	50.3
Bio_3	Isothermality	11.6
Bio_1	Annual mean temperature	9.1
Bio_4	Temperature seasonality	8.3

**Table 3:** The suitable range for the dominant environmental variables.

Environmental Variables	Suitable Range	Adaptive Threshold
Bio_16	404.8~654.5 mm	473.6 mm
Bio_1	11.8~17.4 °C	16.2 °C

**Table 4:** Changes in the centroid of *C. yanhusuo*.

Climate Scenarios	Periods	Longitude (°E)	Latitude (°N)	Migration Distance (km)
	Present	112.64	32.56	
SSP126	2050s	112.26	32.97	53.33
SSP126	2090s	111.99	32.71	79.88 (2050s → 2090s)
SSP585	2050s	114.11	33.09	177.59
SSP585	2090s	113.45	33.32	78.96 (2050s → 2090s)

**Table 5:** Correlation coefficients between THP and environmental variables.

Variables	Tetrahydropalmatine
Mean diurnal temperature range (Bio_2)	0.768 **
Temperature seasonality (Bio_4)	0.842 **
Mean temperature of the wettest quarter (Bio_7)	0.842 **

** Correlation is significant at the 0.01 level (two-tailed).

## Data Availability

Data can be made available upon reasonable request.

## Supplementary Materials

The following supporting information can be downloaded at https://www.mdpi.com/article/10.3390/biology14080972/s1. Table S1. Composition data of active components in *Corydalis yanhusuo* from different geographical origins.
