# Supplementary material for: Prediction Analysis of Integrative Quality Zones for Corydalis yanhusuo W. T. Wang Under Climate Change: A Rare Medicinal Plant Endemic to China
Source: Biology (Basel). 2025 Aug 1;14(8):972. doi: 10.3390/biology14080972 (PMC12383410; doi:10.3390/biology14080972)
Supplement: Supplementary file 1 [file biology-14-00972-s001.zip › biology-3752708-supplementary.pdf]

Supplementary Table S1. Composition data of active components in *Corydalis yanhusuo* from different geographical origins.

Sample	Geographic Origin	Longitude (°E)	Latitude (°N)	Tetrahydropalmatine Content (mg/g)
S1	Xuancheng, Anhui	119.083333	31.050000	0.55
S2	Kaixian, Chongqing	108.350000	31.200000	0.63
S3	Xinyang, Henan	114.733333	32.283333	1.19
S4	Jinyun, Zhejiang	119.983333	28.733333	1.42
S5	Dongyang, Zhejiang	120.366667	29.083333	1.47
S6	Pan'an, Zhejiang	120.383333	28.950000	2.46
S7	Sanhe Town, Chenggu County, Hanzhong	107.355278	33.133889	1.85
S8	Tosha Town, Cheng County, Longnan City, Gansu	105.627778	33.699000	1.05
S9	Pangfeng Village, Guojia Town, Kaixian County, Chongqing	108.423056	31.023056	0.40
S10	Qiqiao Village, Zhangzhi Town, Tongzhou District, Jiangsu	121.021389	31.090833	0.62
S11	Half Kengmen, Renchuan Town, Pan'an County, Zhejiang	120.113611	29.015833	0.71
S12	Shimenmen Township, Kang County, Longnan City, Gansu	105.530833	33.272306	1.44
S13	Experimental field, Sizhou Village, Fuyang District, Hangzhou City, Zhejiang	119.895556	30.083611	0.80
S14	Experimental Cultivation Base, Bai'an Campus, Three Gorges College, Chongqing	108.451667	30.073889	0.50
S15	Miaozigou, Wangbao Village, Luhe Township, Xihe County, Longnan City, Gansu	105.369167	33.964250	0.96
S16	Dongjiaying Village, Dongjiaying Township, Chenggu County, Hanzhong City, Shaanxi	107.263333	33.083611	0.86
S17	Gujhu Village, Xinwu Township, Pan'an County, Zhejiang	120.390833	28.083889	0.42
S18	Unity Village, Zhoujiasan Township, Mianxian County, Hanzhong City, Shaanxi	106.716944	33.014722	0.51
S19	Half Kengmen, Renchuan Town, Pan'an County, Zhejiang	120.440833	28.086389	0.37
S20	Xiaokengmen, Shanghu Town, Pan'an County, Zhejiang	120.640000	29.016111	0.59
S21	Miaoyu Village, Huaqiao Town, Wuhu County, Anhui	118.617222	31.020278	0.44
S22	Fengquan Village, Throwing Sha Town, Cheng County, Longnan City, Gansu	105.627778	33.082222	1.50
